# Psychometric assessment of the Chinese version of the perceptions of palliative care instrument in advanced cancer patients: a cross-sectional study

**DOI:** 10.7717/peerj.20622

**Published:** 2026-01-15

**Authors:** Liping Jiang, Shangjin Li, Kaili Liu, Shanshan Cong, Shudan Zheng, Shaojie Zhao, Bing Zhang

**Affiliations:** 1 Department of Gynaecology, Wuxi Maternal and Child Health Care Hospital, The Affiliated Women’s Hospital of Jiangnan University, Wuxi, Jiangsu, China; 2 Xinxiang Medical University, Xinxiang, China

**Keywords:** Palliative care, Perceptions, Needs, Advanced cancer, Psychometrics, Reliability, Validity

## Abstract

**Background:**

This study aimed to validate the Chinese version of the perceptions of palliative care instrument (C-PPCI) for assessing the perceptions and needs of advanced cancer (AC) patients regarding palliative care in China.

**Methods:**

The C-PPCI was translated following Brislin’s guidelines and tested for psychometric properties through a cross-sectional survey of 537 AC patients. Internal consistency was evaluated using Cronbach’s alpha, and test-retest reliability was assessed with the intra-class correlation coefficient (ICC). Content validity was examined with the content validity index (CVI), and construct validity was explored using exploratory factor analysis (EFA) and confirmed with confirmatory factor analysis (CFA). Concurrent validity was assessed by correlating the C-PPCI with the Edmonton symptom assessment scale (ESAS) and distress thermometer (DT).

**Results:**

Of 537 recruited participants, 444 completed the questionnaire (response rate: 82.6%). The scale-level content validity index (S-CVI) was 0.99. The C-PPCI demonstrated strong internal consistency, with a Cronbach’s alpha of 0.852 for the total scale (subscale range: 0.820–0.872). Test-retest reliability was good, with an ICC of 0.855 for the total scale (subscale range: 0.751–0.815). EFA yielded a four-domain, nine-factor structure explaining 64% to 75% of the total variance. CFA supported this model with good fit indices (comparative fit index (CFI) = 0.917, Tucker-Lewis index (TLI) = 0.904, root mean square error of approximation (RMSEA) = 0.051, standardized root mean square residual (SRMR) = 0.072). Concurrent validity was supported by significant correlations with the ESAS and DT for most subscales.

**Conclusions:**

The 35-item C-PPCI is a valid and reliable instrument for measuring Chinese AC patients’ perceptions and needs regarding palliative care, providing a valuable tool for clinicians to enhance palliative care (PC) utilization in this context.

## Introduction

Palliative care (PC) is to improve quality of life for both patients and their family members facing life-threatening illness by preventing or relieving physical, psychological, social and spiritual sufferings (Kaasa et al., 2018). Living with advanced cancer profoundly impacts a patient’s mental health and body perception. Key issues include altered body image post-treatment, the need for self-compassion due to physical decline, and emotional adjustment challenges. Understanding patients’ views on palliative care, which addresses these issues, is crucial for its effective use. Over the past decade, significant progress has been made in China’s palliative care (PC) landscape, including policy development, service centers and wards, training programs, and academic organizations (Ning, 2018). Despite this, PC development remains slow, with only 0.7% of hospitals offering specialized palliative care services (SPCS) in 2016 (Fang et al., 2020), amid rapid population aging. Key barriers include financial shortages, professional deficits, and misperceptions (Hu & Feng, 2016). Addressing negative attitudes toward PC—among professionals, caregivers, and patients—is essential to identify misunderstandings and promote integration (Hu & Feng, 2016).

Evidence supports early PC engagement for relieving physical symptoms and mental distress, providing spiritual support, and improving quality of life in advanced cancer (AC) patients (Quinn et al., 2020). However, referrals are often delayed due to stigma associating PC with death and hopelessness, persisting across cultures—even in well-established European systems (Zimmermann et al., 2016)—and more profoundly in China (Gu et al., 2016). Late referrals occur even at advanced facilities like Fudan University Affiliated Cancer Hospital, which has provided hospice services since 2006 (Ning, 2018). Timely assessment of patients’ PC attitudes is thus critical for early integration into mainstream healthcare (Gu et al., 2016).

Patients’ PC attitudes are malleable (Perry et al., 2021), prompting educational interventions, though progress remains insufficient and more programs are needed. Existing instruments primarily assess perceptions among professionals (Heydari et al., 2019; Al-Ansari et al., 2019), nurses (Cerratti et al., 2020), students (Sandsdalen et al., 2015), and caregivers (Shah et al., 2020), with few from patients’ perspectives (Zimmermann et al., 2016). The perceptions of palliative care instrument (PPCI), developed in 2013, is the first valid tool capturing AC patients’ attitudes, beliefs, and needs across emotional (positive/negative), cognitive (hopeless, supported, disrupted), needs (emotional/practical/total), burden, and readiness domains (Milne et al., 2013). It remains widely used (Ufere et al., 2019; Hrustanovic-Kadic, Ziegler & El-Kersh, 2021). In comparison, the recent palliative care attitude scale (PCAS-9) (Perry et al., 2020) offers low cognitive burden and theory-based subscales (emotional, cognitive, behavioral) but lacks comprehensive PC consultation perceptions. A 2020 self-designed 16-item questionnaire (Chosich et al., 2020) is clinically grounded and multidimensional but limited to content/face validity. In China’s emerging in-hospital PC context (Qu et al., 2018), PPCI is better suited for initial encounters than PCAS-9.

Interventions’ effectiveness in improving PC knowledge and attitudes depends on meeting patients’ needs (Perry et al., 2021), necessitating joint assessment. Tools like the Chinese Problems and Needs in Palliative Care questionnaire-short version (PNPC-sv) (Wang et al., 2019) multidimensionally evaluate AC needs (physical, psychological, social, spiritual, daily activities, autonomy, financial, informational) (Wang et al., 2018). However, no validated measure combines perceptions and unmet needs from patients’ perspectives in China; PPCI offers a solution to this gap. The PPCI was selected over the other instruments due to its multidimensional assessment of attitudes and needs and its specific suitability for capturing the initial reactions of patients new to palliative care. This is highly relevant in the Chinese context of this study, where many patients with advanced cancer are encountering the concept for the first time (Fang et al., 2020).

This study’s aims were: (1) to translate and culturally adapt the English PPCI into Chinese (C-PPCI); (2) to evaluate its reliability and validity among Chinese AC patients. We hypothesized that the C-PPCI would demonstrate good psychometric properties, including internal consistency, test-retest reliability, content validity, construct validity, and concurrent validity.

## Methods

### Human studies

The present study was established according to the ethical guidelines of the Helsinki Declaration and was approved by the Institutional Review Boards and Ethics Committee of Xinxiang Medical University (XXLL-2020B008).

### Study design

This cross-sectional study validated the reliability and validity of the Chinese version of the perceptions of palliative care instrument (PPCI) using Brislin’s translation guidelines and established cross-cultural adaptation principles. Convenience sampling was employed for participant recruitment, with trained research assistants approaching potential participants in the inpatient wards and outpatient clinics of oncology and related departments. The assistants provided a detailed explanation of the study’s purpose and procedures, screened individuals against inclusion and exclusion criteria, and obtained written informed consent from those who agreed to participate. Data were collected *via* self-administered questionnaires, with assistance from trained investigators as needed.

### Translation and cultural adaptation procedures

The PPCI was translated and culturally adapted into Chinese following Brislin’s guidelines and the cross-cultural adaptation process recommended by the American Academy of Orthopedic Surgeons Evidence-Based Medicine Committee (Jones et al., 2001; Beaton et al., 2000). Specifically, permission to develop the Chinese version of the PPCI (C-PPCI) was first obtained from the original authors and the authors received permission to use this instrument from the copyright holders. Forward translation was performed by two bilingual translators unfamiliar with the PPCI’s concepts and one researcher knowledgeable in palliative care terminology; the preliminary C-PPCI version was synthesized after discussions among the three translators. Back-translation was then conducted by two additional bilingual translators. Cultural adaptation was carried out by a panel of experts, including three palliative care specialists, one linguist, and one psychologist, who convened *via* online conference to evaluate the cultural appropriateness and accuracy of each item. The experts also assessed content validity through a two-round inquiry during this phase. Finally, a pilot test of the C-PPCI was conducted with 30 participants to evaluate its clarity and feasibility.

### Settings and participants

A total of 537 patients with advanced cancer were recruited *via* convenience sampling from Xinxiang Central Hospital in Xinxiang, China, between October 2020 and March 2021. Inclusion criteria were as follows: (1) confirmed diagnosis of advanced-stage cancer, regardless of type; (2) aged 18 years or older; (3) able to understand the study’s purpose; and (4) willing to participate and provide written informed consent. Exclusion criteria included: (1) unconsciousness or disabilities involving literacy, hearing impairment, or inability to communicate; and (2) participation in other interventional studies. The sample size was calculated to be at least 444 participants, accounting for a requirement of 5–10 times the 37 items in the PPCI and an anticipated 20% attrition rate due to missing data. After explaining the study’s objectives, investigators obtained written informed consent from all participants. Data were collected through one-on-one administration, during which investigators distributed the questionnaires and offered assistance as needed; each completed questionnaire was immediately reviewed for completeness to minimize missing data.

### The main measurement instruments

#### The perceptions of palliative care instrument

The original 37-item PPCI was developed by Milne et al. (2013) to assess the attitudes, beliefs, and needs of advanced cancer patients regarding palliative care from their own perspective. It is a self-report instrument comprising four domains: emotional reactions, cognitive reactions, palliative care needs, and perceptions of burden. Items are rated on a 7-point Likert scale, with higher scores indicating more positive perceptions. The PPCI demonstrates strong psychometric properties, comprising four domains and eight subscales: emotional reactions (encompassing positive and negative feelings), cognitive reactions to palliative care (including hopeless, supported, and disrupted perceptions), palliative care needs (emotional, practical, and total needs), and perceptions of burden. Specifically, the emotional reactions domain assesses feelings such as fear, anxiety, depression, stress, reassurance, and hope. The cognitive reactions domain evaluates hopeless, disrupted, and supported responses to PC referrals. A 7-point Likert scale, ranging from 1 (strongly disagree) to 7 (strongly agree), is used for the four domains. For the readiness item, a 10-point Likert scale is employed, from 0 (not at all) to 10 (totally).

#### The edmonton symptom assessment scale

The Edmonton symptom assessment scale (ESAS) is a patient-reported tool used to rate the intensity of nine common cancer-related symptoms: pain, fatigue, nausea, depression, anxiety, drowsiness, appetite, well-being, and shortness of breath. Each symptom is rated on an 11-point numerical scale from 0 (symptom absent) to 10 (worst possible severity). This study used the validated Chinese version of the ESAS, translated by Dong et al. (2015) which has demonstrated good reliability with a Cronbach’s α coefficient of 0.86.

#### Distress thermometer

The distress thermometer (DT), a single-item tool for rapidly assessing psychological distress in cancer patients, was originally developed and published by Roth et al. (1998). It consists of an 11-point scale, analogous to a thermometer, where patients circle a number from 0 (“No distress”) to 10 (“Extreme distress”) that best describes their experience over the last 24 h. We utilized the Chinese version translated by Tang et al. (2011). A score of 4 or higher is typically used as a cutoff to indicate a clinically significant level of distress requiring further assessment.

### Statistical analysis

Data were analyzed using IBM SPSS version 22.0 and Mplus version 7.0, with the statistical significance level set at 0.05. Reliability was assessed *via* Cronbach’s alpha for internal consistency and test-retest reliability. Item analysis encompassed item discrimination, item-total correlations (≥0.30) (Streiner, Norman & Health, 2015), and critical ratio values (CR > 3.0) (Mosier & Mcquitty, 1940). Furthermore, item analysis extended beyond statistical metrics to incorporate theoretical and practical considerations (Hu, Leeuwen & Li, 2019). Acceptable Cronbach’s alpha values were ≥0.7 (Streiner, Norman & Health, 2015). Test-retest reliability was evaluated using the intraclass correlation coefficient (ICC), based on data from 50 AC patients who completed the questionnaire battery again two weeks later.

Content validity was assessed using the content validity index (CVI), rated by a panel of experts—including three palliative care specialists, one linguist, and one psychologist—on a 4-point Likert scale from 1 (strongly disagree) to 4 (strongly agree). Item-CVI values exceeding 0.7 indicated satisfactory content validity (Grant & Davis, 1997).

Patients’ physical symptoms and emotional distress have been identified as major predictors of cancer patients’ needs (Wang et al., 2018; Wang et al., 2021). Additionally, emotional distress is associated with reduced preferences for PC due to misconceptions and delayed gratification (Gerhart et al., 2016). This explains the selection of the DT and ESAS for evaluating concurrent validity in the original PPCI. Similarly, concurrent validity of the C-PPCI was examined using Pearson’s correlation coefficients between the C-PPCI and both the DT and ESAS. Construct validity of the C-PPCI was evaluated through exploratory factor analysis (EFA). Prior to EFA, sampling adequacy was confirmed using the Kaiser-Meyer-Olkin (KMO) test (>0.6) and Bartlett’s test of sphericity (*p* < 0.001). Factors were extracted based on eigenvalues > 1.0 and factor loadings > 0.40 (Tabachnick & Fidell, 2007). Confirmatory factor analysis (CFA) was performed using Mplus version 7.0, with acceptable model fit criteria including *χ*^2^/df ≤ 3, root mean square error of approximation (RMSEA) ≤ 0.08, comparative fit index (CFI) > 0.90, standardized root mean square residual (SRMR) ≤ 0.08, and Tucker-Lewis index (TLI) > 0.90 (Hu & Bentler, 1999).

## Results

### Demographic characteristics

Of the 537 recruited participants, 444 (82.6%) completed the questionnaire. Their demographic characteristics are summarized in Table 1. The majority of these AC patients were female (53.8%) and married (93.5%), with lung cancer (27.5%) and breast cancer (15.8%) being the most common diagnoses. The subsamples for EFA and CFA exhibited similar demographic profiles (Table 1). Although not precisely measured, the average completion time ranged from 10 to 20 min, suggesting the feasibility of implementing the C-PPCI among AC patients.

**Table 1:** Demographic characteristics of the total sample (*N* = 444) and subsamples for exploratory factor analysis (EFA) and confirmatory factor analysis (CFA).

		Total sample		EFA sample		CFA sample	
		N (444)	%	N (222)	%	N (222)	%
Age (Mean ± SD)		61.39 ± 11.68		61.59 ± 11.30		61.25 ± 12.09	
Gender	Male	205	46.2	93	41.9	112	50.4
	Female	239	53.8	129	58.1	110	49.6
Educational level	Illiteracy	42	9.5	24	10.8	18	8.1
	Primary school	116	26.1	66	29.7	50	22.5
	Junior school	154	34.7	73	32,9	81	36.5
	High school	118	26.6	54	24.3	64	28.8
	University	14	3.2	5	2.3	9	4.1
Living area	Urban	217	48.9	100	45.5	117	52.7
	Rural	227	51.1	122	55.5	105	47.3
Financial pressure	Non	87	19.6	42	18.9	45	20.3
	Yes	357	80.4	180	81.1	177	79.7
Clinical stage	III	247	55.6	131	59.0	116	52.3
	IV	197	44.4	91	41.0	106	47.7
Cancer site	Lung caner	122	27.5	57	25.7	65	29.3
	Breast cancer	70	15.8	35	15.8	35	15.8
	Gastric cancer	47	10.6	17	7.7	30	13.5
	Liver cancer	24	5.4	11	5.0	13	5.9
	Colorectal cancer	41	9.2	25	11.3	16	7.2
	Other cancer	140	31.5	77	34.7	63	28.4
Operation	No	219	49.3	110	49.5	109	49.1
	Yes	225	50.7	112	50.5	113	50.9
Chemotherapy	1∼3 times	143	32.2	72	32.4	71	32.0
	4∼6 times	109	24.5	55	24.8	54	24.3
	More than 7 times	192	43.2	95	42.8	97	43.7

**Notes.**

EFA exploratory factor analysis CFA confirmatory factor analysis SD standard deviation N number of participants % Percentage

### Instrument modification

Based on expert recommendations during the translation and cultural adaptation phase, several modifications were made to enhance the clarity of the C-PPCI items. The panel noted that the PPCI assesses not only emotional and cognitive reactions of advanced cancer (AC) patients upon first learning about palliative care (PC) but also their needs. Consequently, the instrument’s name was revised to “Perceptions of Palliative Care and Needs Instrument” in Chinese to better reflect its scope. For Item 18, experts found the term “strangers” ambiguous and revised it to “strangers associated with providing palliative care” for clarity. To broaden the applicability of the C-PPCI to other terminally ill patients beyond those with AC, Item 9 was modified from “Think my cancer is out of control” to “Think my disease is out of control”. Additionally, since 2016, the terms “hospice”, “palliative care”, and “end-of-life care” have been officially standardized as “Anning Liaohu” in China (Ning, 2018); thus, “palliative care” was translated accordingly in the C-PPCI.

### Item analysis

Table 2 presents the item discrimination results, with critical values exceeding 3.0 for all items. As shown in Table 3, item-total correlations were statistically significant across all items. However, items 2, 4, 16, 17, 20, 21, and 29 exhibited weak correlations. These items were retained in the C-PPCI as their item-total correlations approximated 0.30. Item 18 was excluded due to an item-total correlation substantially below 0.30. Item 7 was retained, despite its lower correlation, to maintain the three items assessing positive emotional reactions in the original PPCI.

**Table 2:** Critical values (t-Statistics) for items in the Chinese version of the perceptions of palliative care instrument (C-PPCI) (*N* = 444).

Item	*t*	*P*	95% confidence interval	
			Lower	Upper
1	10.720	<0.001	1.492	2.163
2	5.777	<0.001	0.697	1.418
3	12.374	<0.001	1.806	2.489
4	5.422	<0.001	0.611	1.307
5	14.582	<0.001	1.870	2.441
6	14.561	<0.001	1.878	2.466
7	3.905	<0.001	0.374	1.135
8	3.638	<0.001	0.305	1.023
9	16.638	<0.001	2.269	2.878
10	3.424	0.001	0.212	0.788
11	15.071	<0.001	2.116	2.752
12	15.924	<0.001	2.148	2.754
13	10.691	<0.001	1.297	1.883
14	17.512	<0.001	2.284	2.863
15	11.650	<0.001	1.505	2.118
16	7.312	<0.001	0.934	1.623
17	8.789	<0.001	1.094	1.726
18	4.363	<0.001	0.450	1.190
19	7.459	<0.001	0.977	1.679
20	7.662	<0.001	1.041	1.762
21	2.701	0.007	0.238	1.517
22	9.427	<0.001	1.686	2.576
23	6.819	<0.001	1.201	2.176
24	7.582	<0.001	1.213	2.065
25	4.176	<0.001	0.472	1.315
26	3.181	0.002	0.240	1.022
27	8.572	<0.001	1.357	2.167
28	8.192	<0.001	1.301	2.125
29	2.877	0.004	0.186	0.994
30	5.977	<0.001	0.764	1.515
31	6.655	<0.001	0.889	1.636
32	9.295	<0.001	1.544	2.374
33	4.403	<0.001	0.503	1.317
34	6.960	<0.001	0.934	1.672
35	11.995	<0.001	2.185	3.044
36	15.628	<0.001	2.400	3.092
37	12.208	<0.001	2.190	3.027

**Notes.**

C-PPCI Chinese version of the perceptions of palliative care instrument N number of participants t t-statistic P *p*-value

**Table 3:** Item-content validity index (I-CVI) and item-total correlations for the Chinese version 2of the perceptions of palliative care instrument (C-PPCI) (*N* = 444).

Item	I-CVI	Item-total correlation
1. I feel scared	1	0.332^**^
2. I feel hopeful	1	0.287^**^
3. I feel stressed	1	0.379^**^
4. I feel secure	1	0.282^**^
5. I feel depressed	1	0.400^**^
6. I feel anxious	1	0.410^**^
7. I feel reassured	1	0.197^**^
8. Think the more support I get the better I will feel	1	0.318^**^
9. Think my illness is out of control	1	0.458^**^
10. Think my doctor really cared about what is happening to me	1	0.306^**^
11. Think nothing more can be done	1	0.439^**^
12. Think I am at the end of the road	1	0.464^**^
13. Think my doctor has given up on me	1	0.377^**^
14. Think my illness is terminal	1	0.488^**^
15. Think I will lose contact with my current doctors and nurses	1	0.368^**^
16. Think about the future more positively	1	0.295^**^
17. Feel more in control of my situation	1	0.292^**^
18. See this as having strangers associated with providing palliative care coming into my home	0.800	0.216^**^
19. Be worried that they would disrupt my daily routine	1	0.325^**^
20. Worry that they would talk to me about dying	1	0.245^**^
21. How ready are you to hear about palliative care now?	1	0.151^**^
22. My family or friends need help with my physical care	1	0.486^**^
23. I need help to manage physical symptoms such as pain	0.800	0.382^**^
24. I need emotional support	1	0.489^**^
25. I need help to manage my medication	1	0.361^**^
26. I want to talk with someone about dying	1	0.303^**^
27. My family or friends need emotional support	1	0.487^**^
28. I need spiritual support	1	0.489^**^
29. I want someone to talk to my family or friends about my illness	1	0.257^**^
30. I want help to find meaning in my illness experience	1	0.345^**^
31. I want to prepare now for what might happen in the future	1	0.335^**^
32. I need help with my daily activities such as showering	1	0.477^**^
33. I want to talk to someone who understands what I am going through	1	0.303^**^
34. I want my family and friends to prepare now for what might happen in the future	1	0.380^**^
35. I worry I am a burden to others	1	0.505^**^
36. I feel I depend too much on others to do things for me	1	0.566^**^
37. I worry that my family and friends don’t have enough time for themselves because of what they do for me	1	0.533^**^

**Notes.**

** *P* < 0.001.

I-CVI item-content validity index N number of participants P *p*-value

### Content validity

As presented in Table 3, the scale-level content validity index (S-CVI) for the C-PPCI was 0.99. The item-level content validity index (I-CVI) was 1.0 for all items except items 18 and 23, which had an I-CVI of 0.80. All CVI values met or exceeded 0.8, indicating strong content relevance for the C-PPCI (Grant & Davis, 1997).

### Construct validity

Consistent with the original PPCI, we employed the same analytical approach to assess the construct validity of each domain in the C-PPCI separately (Milne et al., 2013). The data were suitable for principal component analysis, as evidenced by Kaiser-Meyer-Olkin (KMO) values ranging from 0.660 to 0.860 across the four domains and a significant Bartlett’s test of sphericity (*p* < 0.001). Similar to the original PPCI, exploratory factor analysis (EFA) identified four domains—emotional reactions, cognitive reactions, palliative care needs, and perceptions of burden—explaining 64% to 75% of the total variance per domain, as shown in Table 4. EFA results revealed nine factors with eigenvalues greater than 1.0 and factor loadings exceeding 0.4. Notably, the palliative care needs domain diverged from the original PPCI based on statistical criteria.

**Table 4:** Results of exploratory factor analysis (EFA) for the Chinese version of the perceptions of palliative care instrument (C-PPCI) (*N* = 222).

Domain	Factor	Items	Factor loading	KMO	Variance explained
Emotional reactions to PC	Negative feelings	ppci5	0.913	0.791	44.450
		ppci3	0.880		
		ppci6	0.865		
		ppci1	0.806		
	Positive feelings	ppci4	0.866		31.009
		ppci2	0.835		
		ppci7	0.769		
Cognitive reactions to PC	Hopeless	ppci12	0.856	0.860	32.732
		ppci11	0.830		
		ppci14	0.829		
		ppci9	0.800		
		ppci13	0.648		
		ppci15	0.615		
	Supported	ppci8	0.753		16.057
		ppci10	0.738		
		ppci16	0.514		
	Disrupted	ppci17	0.457		15.075
		ppci20	0.816		
		ppci19	0.807		
Palliative care needs	Emotional and spiritual	ppci28	0.864	0.747	20.326
		ppci27	0.858		
		ppci24	0.858		
	Physical needs	ppci32	0.901		18.216
		ppci22	0.868		
		ppci23	0.730		
	Communication needs	ppci29	0.810		17.032
		ppci30	0.792		
		ppci33	0.779		
	Preparations for the future	ppci31	0.873		16.145
		ppci34	0.823		
		ppci26	0.621		
Perceptions of burden		ppci35	0.923	0.660	75.443
		ppci37	0.889		
		ppci36	0.788		

**Notes.**

EFA exploratory factor analysis PC palliative care KMO Kaiser-Meyer-Olkin measure of sampling adequacy N number of participants

Specifically, items 22, 23, and 32, related to physical management (*e.g.*, physical care, showering, and pain management), formed a factor labeled “physical needs”, accounting for 18.21% of the variance in the PC needs domain. Items 24, 27, and 28, pertaining to emotional or spiritual support, were grouped as “emotional and spiritual needs”, explaining 20.33% of the variance. Items 29, 30, and 33, focused on discussions about the illness, were categorized as “communication needs”, contributing 17.07% to the variance. Lastly, items 26, 31, and 34, addressing preparations for the future or conversations about dying, were labeled “preparation needs for the future”, accounting for 16.15% of the variance. Beyond statistical criteria, logical judgment led to the removal of item 25, as it was unrelated to other items in the emotional and spiritual needs factor.

As presented in Table 5, confirmatory factor analysis (CFA) for each subscale confirmed acceptable fit indices: *χ*^2^/df ranged from 0.000 to 2.289, RMSEA from 0.000 to 0.076, CFI from 0.941 to 1.000, TLI from 0.922 to 1.000, and SRMR from 0.000 to 0.055. Improved fit indices supported the removal of item 25 (Table 5). Overall, CFA validated a nine-factor model with robust fit indices: *χ*^2^/*df* = 1.574, RMSEA = 0.051, CFI = 0.917, TLI = 0.904, and SRMR = 0.072.

### Concurrent validity

As shown in Table 6, significant positive correlations were observed between the DT and emotional reactions (*r* = 0.104, *p* = 0.029), palliative care needs (*r* = 0.117, *p* = 0.014), and perceptions of burden (*r* = 0.267, *p* < 0.001). However, no significant correlation was found between the DT and cognitive reactions (*r* = 0.000, *p* = 0.996). Similarly, positive correlations were identified between the ESAS and emotional reactions (*r* = 0.107, *p* = 0.024), palliative care needs (*r* = 0.279, *p* < 0.001), and perceptions of burden (*r* = 0.407, *p* < 0.001). No significant association was detected between ESAS and cognitive reactions (*r* = 0.064, *p* = 0.307).

### Internal consistency reliability, test-retest reliability

As presented in Table 7, the Cronbach’s alpha coefficient for the total C-PPCI was 0.852, with values ranging from 0.820 to 0.872 for the four subscales, indicating robust internal consistency reliability. To evaluate test-retest reliability, 50 patients with AC completed the C-PPCI again after two weeks. The intraclass correlation coefficient (ICC) was 0.855 for the total scale and ranged from 0.751 to 0.815 for the subscales, demonstrating strong stability over time.

**Table 5:** Model fit indices from confirmatory factor analysis (CFA) for the Chinese version of the perceptions of palliative care instrument (C-PPCI) (*N* = 222).

Items	*χ*^2^/*df*	*RMSEA*	*CFI*	*TLI*	*SRMR*
Emotional reactions	2.289	0.076	0.973	0.956	0.052
Palliative care needs with Item 25	1.814	0.061	0.953	0.93.8	0.059
Palliative care needs without Item 25	1.553	0.050	0.973	0.963	0.055
Perceptions of Burden	<0.001	<0.001	1.000	1.000	<0.001
Total scale without Item 25	1.574	0.051	0.917	0.904	0.072

**Notes.**

CFA Confirmatory Factor Analysis *χ*2/*df* Chi-square divided by degrees of freedom CFI comparative fit index TLI Tucker-Lewis Index RMSEA root mean square error of approximation SRMR standardized root mean square residual N number of participants

**Table 6:** Correlations for concurrent validity of the Chinese version of the perceptions of palliative care instrument (C-PPCI) with the Edmonton symptom assessment scale (ESAS) and 3distress thermometer (DT) (*N* = 444).

	Total scale	Emotional reactions	Cognitive reactions	Palliative care needs	Perceptions of Burden
ESAS	0.308^**^	0.107^*^	0.064	0.279^**^	0.407^**^
DT	0.156^**^	0.104^*^	<0.001	0.117^*^	0.267^**^

**Notes.**

* *P* < 0.05.

** *P* < 0.001.

ESAS Edmonton symptom assessment scale DT distress thermometer N number of participants P *p*-value

**Table 7:** Internal consistency (Cronbach’s Alpha) and test-retest reliability of the Chinese version of the perceptions of palliative care instrument (C-PPCI) (*N* = 444).

Items	Cronbach’s α	Test-retest reliability
Total scale	0.852	0.855^**^
Emotional reactions	0.849	0.815^**^
Cognitive reactions	0.872	0.806^**^
Palliative care needs	0.820	0.814^**^
Perceptions of burden	0.833	0.751^**^

**Notes.**

** *P* < 0.001.

N Number of participants P *p*-value

## Discussion

The 37-item Perceptions of Palliative Care Instrument (PPCI) was the first validated tool to comprehensively assess beliefs, attitudes, and needs related to palliative care (PC) from the perspective of patients with advanced cancer (AC). It is particularly well-suited for AC patients encountering PC for the first time. As PC services become increasingly available in China, identifying and addressing misperceptions about PC is crucial for overcoming barriers and promoting timely utilization. Therefore, this study aimed to translate the PPCI into Chinese (C-PPCI) and evaluate its psychometric properties. The findings demonstrated strong validity, reliability, and acceptability of the C-PPCI, establishing it as, to our knowledge, the first robust measure in China to assess attitudes, needs, and self-perceived burdens associated with PC.

Regarding item analysis, CR values for all 37 items met the requirements for item differentiation, except for items 21 and 29, which were near the threshold and thus retained in the C-PPCI. Weaker item-total correlations were observed, likely due to the multidimensional nature of the C-PPCI. Consistent with the original PPCI, item 21 exhibited low item-total correlation, possibly reflecting the limitations of a single-item measure (Milne et al., 2013). The readiness item, “How ready are you to hear about PC now?” likely captures a non-rejective attitude, representing an initial step toward discussing or accepting PC referral. Although validated instruments exist to assess home care staff preparedness for PC (Chan et al., 2018) or professionals’ knowledge and self-efficacy in PC preparation (Phillips, Salamonson & Davidson, 2011; Gupta et al., 2023), to our knowledge, no validated tool evaluates readiness to discuss PC from the patient’s perspective (Sherman et al., 2018). Therefore, retaining this readiness item was deemed necessary despite its suboptimal item-total correlation (Perry et al., 2021).

The EFA results indicated that items in each subscale accounted for 64% to 75% of the total variance. The three domains of the C-PPCI—emotional reactions, cognitive reactions, and perceptions of burden—were fully consistent with those of the original PPCI. Moreover, the total variance explained by these three domains in the C-PPCI was comparable to that of the original PPCI, suggesting structural similarity between the two instruments. Notably, the palliative care needs subscale in the C-PPCI explained 71.72% of the total variance, surpassing the 63.9% in the original PPCI subscale (Milne et al., 2013). This difference may reflect the more detailed needs captured by the C-PPCI, encompassing physical needs, emotional and spiritual needs, communication needs, and preparedness needs for the future, compared to the original PPCI’s broader categories of emotional, practical, and total needs. CFA confirmed a four-domain, nine-factor model with robust fit indices.

The emotional and cognitive reaction domains of the C-PPCI capture both negative and positive responses to PC referral. Emotions toward PC services, particularly upon initial exposure, are complex and cannot be accurately assessed using a single dimension. Compared to the PCAS-9, which primarily focuses on stressful reactions, the C-PPCI offers a more comprehensive assessment of holistic responses to PC (Perry et al., 2020). Such thorough evaluation is essential, especially as efforts to promote PC services in China have intensified since the implementation of PC pilot programs (Fang et al., 2020). Our findings highlight the importance of holistically assessing emotional and cognitive reactions from the patient’s perspective to objectively understand attitudes toward PC purpose.

Notably, the needs domain in the C-PPCI expanded to four detailed components, compared to the three-component emotional needs in the original PPCI. Approximately 70% of patients identified pain as the primary unmet need among the physical needs of Chinese AC patients (Wang et al., 2021), consistent with prior PC needs instruments (Wang et al., 2019). The physical needs factor was rigorously extracted and validated in the C-PPCI. Item retention decisions considered both statistical and theoretical logic. Item 25, “I need help to manage my medication”, was deemed logically unrelated to other items (Dong et al., 2015; Mosier & Mcquitty, 1940; Hu, Leeuwen & Li, 2019) and was excluded from the emotional and spiritual needs factor. This exclusion increased the proportion of variance explained in the PC needs domain from 69.5% to 71.7% and improved fit indices for the nine-factor C-PPCI model, confirming the appropriateness of removing item 25. Based on EFA and CFA results, a factor labeled “communication needs” emerged from items 29, 30, and 33, capturing AC patients’ desire for empathetic discussions with professionals about their illness and its meaning, either with themselves or their family members. The majority of participants in this study were older cancer patients, and prior research indicates that most older patients are information seekers or listeners, with only 14% avoiding information related to PC (Bol et al., 2020). Additionally, 68% of breast cancer patients expressed interest in further discussions after reading a brief WHO definition of PC, suggesting a need for communication and a somewhat favorable attitude toward PC (Yan et al., 2020).

The needs of AC patients are complex, multifaceted, and vary by context (Wang et al., 2018). Unlike the PC needs in the original PPCI, items 26, 31, and 34 in the C-PPCI formed a new factor labeled “preparedness needs for the future”, strongly supported by EFA and CFA results. This likely reflects Chinese cultural taboos around death, where expressions of “preparing for death” are often implicit (Wang et al., 2023). Advance preparation is considered a critical component of a good death, underscoring the importance of providers addressing this from the palliative patient’s perspective (Kastbom, Milberg & Karlsson, 2017). Consistent with the benefits of early PC integration, proactive preparation for the future offers AC patients opportunities to develop contingency plans for end-of-life care, alleviating significant distress (Hannon et al., 2017). Thus, the “preparedness needs for the future” factor is a compelling and essential element of the C-PPCI.

Additionally, including the perceptions of burden subscale in the C-PPCI is well-justified. Self-perceived burden among AC patients is associated with depression (Karabuga Yakar et al., 2023) and, in some cases, a desire for hastened death, warranting early PC intervention (Gudat et al., 2019). Consistent with prior studies, our findings revealed a positive correlation between perceived burden and DT scores, indicating that greater perceived burden corresponds to increased distress. Aligning with original PPCI results (Milne et al., 2013), accumulating evidence shows that PC services, particularly early integrated models, provide effective coping support (Greer et al., 2020). Assessing the extent to which AC patients perceive their health status as a burden to others can serve as a catalyst for seeking PC assistance. Both caregiver burden and self-perceived burden increase the preference for PC services (Kuharic et al., 2025). Perceptions of burden reflect dependency and unmet needs (Rodríguez-Prat et al., 2019), and timely assessment can dynamically highlight these unmet needs to some extent. Significant associations between with DT, ESAS and C-PPCI indicated acceptable concurrent validity of C-PPCI. In line with PPCI, no correlations were found between DT, ESAS, and cognitive reactions. It might be related to the nature that cognitive reactions merely referred to beliefs about PC referral (Milne et al., 2013). Some of Cronbach alpha were more excellent in the original PPCI than those in C-PPCI, but all of Cronbach alpha in C-PPCI were more than 0.8, indicating excellent internal reliable (Goyarrola et al., 2024). Robust test-retest reliability suggested good temporal stability and reproducibility of C-PPCI.

### Strength and limitations

A key strength of this study is that the C-PPCI was established as a valid, reliable, and multifaceted tool for assessing AC patients’ perceptions, needs, and self-perceived burdens related to PC, using a concise set of items. It is particularly well-suited for patients encountering PC for the first time. Additionally, the development of the C-PPCI addresses a critical gap by providing a robust measure tailored to the Chinese cultural context, while also facilitating cross-cultural comparisons.

The primary limitation is that participants were recruited from a single center in Henan without integrated PC services. Chosich reported that cancer patients exposed to PC or familiar with it tend to harbor fewer negative emotions toward it (Chosich et al., 2020). Future studies should validate the C-PPCI in patients who have received PC services, encompassing both cancer and other life-threatening chronic conditions. PC services remain incompletely integrated into China’s mainstream healthcare system (Willemsen et al., 2021), and while home-based PC services are currently limited yet in high demand (Liu et al., 2021), future research could explore the C-PPCI’s application in home-based settings to promptly identify and address barriers to PC utilization. Furthermore, the integration of artificial intelligence (AI) and machine learning (ML) presents a promising frontier for enhancing patient-centered palliative care. AI-driven tools could potentially analyze patient-reported data from instruments like the C-PPCI in real-time to predict unmet needs, personalize symptom management plans, and facilitate more timely and effective communication between patients and providers (Dixon et al., 2024). Addressing these human-centered challenges through technology could help scale the benefits of palliative care, a crucial consideration in a large and resource-diverse country like China. For example, ML models could identify patient clusters with specific perception and need profiles, allowing for targeted educational interventions (Thacharodi et al., 2024). The C-PPCI could serve as a valuable data source for developing and validating such intelligent healthcare systems in the future.

## Conclusions

The 35-item C-PPCI is a robust and comprehensive tool for assessing perceptions, needs, and self-perceived burdens related to PC, particularly well-suited for patients encountering PC services for the first time. Its development facilitates the identification of barriers to PC from the patient’s perspective, supports the evaluation of educational interventions aimed at correcting misconceptions about PC, and ultimately promotes the utilization and advancement of PC services in China.

##  Supplemental Information

10.7717/peerj.20622/supp-1 Supplemental Information 1 Original Raw Data

10.7717/peerj.20622/supp-2 Supplemental Information 2 Questionnaire (Original)

10.7717/peerj.20622/supp-3 Supplemental Information 3 Questionnaire (English)

## Abbreviations

AC: Advanced cancer
C-PPCI: Chinese version of Perceptions of Palliative Care Instrument
CFA: Confirmatory factor analysis
CFI: Comparative fit index
DT: Distress thermometer
EFA: Exploratory factor analysis
ESAS: Edmonton Symptom Assessment Scale
I-CVI: Item-content validity index
PC: Palliative care
PPCI: Perceptions of Palliative Care Instrument
RMSEA: Root mean square error of approximation
SPCS: Specialized palliative care service
SRMR: Standardized root mean square residual
TLI: Tucker-Lewis Index.

## Additional Information and Declarations

### Data Availability

The following information was supplied regarding data availability: The raw measurements are available in the Supplementary Files.
