# Supplementary material for: Psychometric assessment of the Chinese version of the perceptions of palliative care instrument in advanced cancer patients: a cross-sectional study
Source: PeerJ. 2026 Jan 15;14:e20622. doi: 10.7717/peerj.20622 (PMC12812269; doi:10.7717/peerj.20622)
Supplement: Supplemental Information 2 [file peerj-14-20622-s002.pdf]

一般资料问卷

1.姓名：________________ 2.电话：________________

3.年龄： ________________4.性别： ① 男 ②女

5.身高：______cm 体重：______ kg

6.民族：①汉族 ②少数民族 _________

7.宗教信仰：①无 ②有 _________

8.参加宗教活动的次数：①每周一至两次 ②每周三到四次

9.婚姻状况: ①未婚 ②已婚 ③丧偶 ④离异 ⑤其他

10.文化程度: ①不识字 ②小学 ③初中 ④高中及大专 ⑤本科及以上学历

11.您曾经/现在的职业是：①农民 ②教师③工人④医生 ⑤公务员 ⑥自由职业 ⑦其他

12.您的居住方式：① 独居 ②与家人/朋友一起生活 ③辅助机构或疗养机构 ④其他

13.您的居住地：①城镇 ②农村

14.您的月收入大约：①＜1000 ②1000～ ③2000～ ④3000～ ⑤4000～ ⑥≥5000

15.您是否有经济压力：①无 ②有

16.您的医疗费用情况：①合作医疗 ②自费 ③公费医疗 ④商业医疗保险 ⑤其他

17.癌症类型：①肺癌 ②肝癌 ③胃癌 ④结直肠癌 ⑤乳腺癌 ⑥其他 _________

18.您是否接受过手术治疗：①否 ②是

19.您从确诊疾病到本次调查的时间： ___________

20.您住院化疗次数：①1~3 次 ②4~6 次 ③7 次以上

21.您在化疗期间是否出现过不良反应：

①无 ②恶心/呕吐 ③贫血/出血 ④手脚麻木 ⑤脱发 ⑥其他 _________

22.您在治疗期间是否接受过临床药物试验：①否 ②是

23.疾病分期：①I期 ②II期 ③III期 ④IV期

24.您是否有除癌症之外的其他疾病：①否 ②是： __________

25.病情知晓情况：①不了解 ②有一点了解 ③基本了解 ④完全了解

26.您是否了解安宁疗护：①从未了解过 ②只了解一点点 ③了解并能够解释

27.您了解安宁疗护信息的渠道：①杂志/书籍 ②手机网络 ③其他社交媒体

安宁疗护感知与需求量表

说明：请在每个条目后您当下最认同的观点对应的数字上画✓。回答没有对错之分，我们对您当下的看法很感兴趣。

当我听到安宁疗护时，当下会对以下说法有多大程度上的认同?							
	非常不同意	不同意	稍微不同意	既不同意也不反对	稍微同意	同意	非常同意
1.我感到害怕							
2.我感到有希望							
3.我感到有压力							
4.我感到有安全感							
5.我感到沮丧							
6.我感到焦虑							
7.我感到很舒心							
如果现在医生建议我转诊安宁疗护的话，我会......							
	非常不同意	不同意	稍微不同意	既不同意也不反对	稍微同意	同意	非常同意
8.我觉得获得的支持越多感觉越好							
9.我觉得我的疾病已无法控制							
10.我觉得医生真的很关心我							
11.我觉得没有其他可尝试的了							
12.我觉得我已经走到了生命的尽头							
13.我觉得我的医生已经放弃我了							
14.我觉得我的病已经是绝症了							
15.我觉得我将与当前治疗我的医生、护士失去联系							
16.我会更积极的看待未来							
17.我觉得能更好得掌控自己的状态							
18.我觉得可能将有“陌生的安宁疗护团队的人”来到我家							
19.我会担心他们会扰乱我的日常生活							
20.我会担心他们要和我谈论死亡							

21. 现在来了解下安宁疗护，您准备好了吗？										
0	1	2	3	4	5	6	7	8	9	10
完全没有准备好										完全准备好

以下需求属于安宁疗护服务范畴，是可以帮助到您、您的家人及朋友，请评估自己当下的需求和状态							
	非常不同意	不同意	稍微不同意	既不同意也不反对	稍微同意	同意	非常同意
22.我的家人或朋友需要帮我进行身体照护							
23.我需要帮助来控制身体症状，比如疼痛							
24.我需要情感支持							
25.我需要在用药管理方面得到帮助							
26.我想跟人谈谈死亡的事							
27.我的家人或朋友需要情感支持							
28.我需要精神或灵性支持							
29.我希望有人跟我的家人或朋友谈谈我的病情							
30.患了疾病后，我想要别人帮助以探寻疾病经历的意义							
31.我想为将来可能发生的事情做准备							
32.我需要别人照料我的日常生活，比如洗澡							
33.我想和一个能理解我正在经历什么的人聊聊							
34.我希望我的家人和朋友能够为将来发生的事情有所准备							
当我想到目前的健康状况时，现在会对以下说法有多大程度上的认同?							
	非常不同意	不同意	稍微不同意	既不同意也不反对	稍微同意	同意	非常同意
35.我担心我是别人的负担							
36.我觉得我太依赖别人照顾我了							
37.我担心我的家人和朋友因为照顾我而没有属于自己的时间							

埃德蒙顿症状评价系统（ESAS）

请圈出最能描述在最近 24 小时中您自己的健康状态的数字

心理痛苦温度计（DT）

首先，请在最符合您近一周所经历的平均痛苦水平的数字上“✓”

无疼痛	0	1	2	3	4	5	6	7	8	9	10	极度疼痛
不疲倦	0	1	2	3	4	5	6	7	8	9	10	极度疲倦
不恶心	0	1	2	3	4	5	6	7	8	9	10	极度恶心
不抑郁	0	1	2	3	4	5	6	7	8	9	10	极度抑郁
不焦虑	0	1	2	3	4	5	6	7	8	9	10	极度焦虑
不瞌睡	0	1	2	3	4	5	6	7	8	9	10	极度瞌睡
食欲极好	0	1	2	3	4	5	6	7	8	9	10	食欲极差
感觉生活质量极佳	0	1	2	3	4	5	6	7	8	9	10	感觉生活质量极差
不骚痒	0	1	2	3	4	5	6	7	8	9	10	极度瘙痒
无气急	0	1	2	3	4	5	6	7	8	9	10	极度气急
其他问题	0	1	2	3	4	5	6	7	8	9	10	

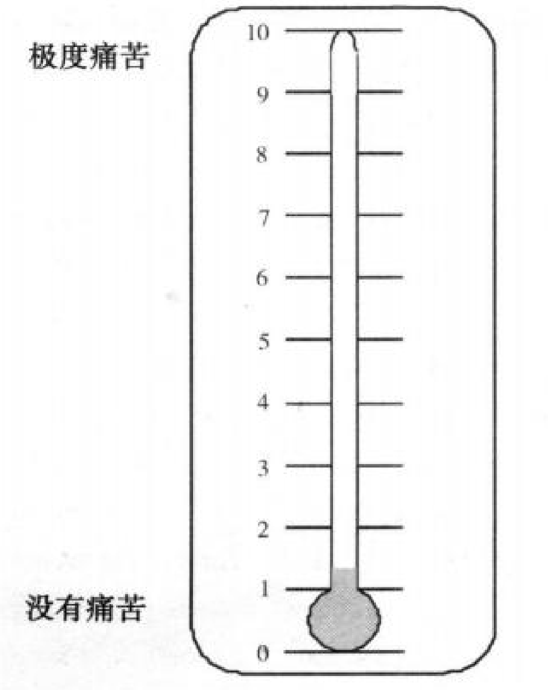

接着，请指出下列哪些选项是引起您痛苦的原因？并在该项目前打“✔”。

	**实际问题**
☐	无时间精力照顾孩子/老人
☐	无时间精力做家务
☐	经济问题
☐	交通出行
☐	工作/上学
☐	周围环境
	**交往问题**
☐	与孩子/老人相处
☐	与伴侣相处
☐	与亲友相处
☐	与医护人员相处
	**情绪问题**
☐	抑郁
☐	恐惧
☐	孤独
☐	紧张
☐	悲伤
☐	担忧
☐	对日常活动丧失兴趣
☐	睡眠
☐	记忆力下降 /注意力不集中

	**身体问题**
☐	外表/形体
☐	洗澡 /穿衣
☐	呼吸
☐	排尿改变
☐	便秘
☐	腹泻
☐	进食
☐	疲乏
☐	水肿
☐	发烧
☐	头晕
☐	消化不良
☐	口腔疼痛
☐	恶心
☐	鼻子干燥/充血
☐	疼痛
☐	性
☐	皮肤干燥
☐	手/脚麻木
☐	身体活动受限制
	**信仰/宗教问题**
☐	信仰/宗教问题
