# Supplementary material for: Psychometric assessment of the Chinese version of the perceptions of palliative care instrument in advanced cancer patients: a cross-sectional study
Source: PeerJ. 2026 Jan 15;14:e20622. doi: 10.7717/peerj.20622 (PMC12812269; doi:10.7717/peerj.20622)
Supplement: Supplemental Information 3 [file peerj-14-20622-s003.pdf]

General Information Questionnaire

1.surname and personal name : ________________ 2. Tel:________________

3.age : ________________4. Gender: ① Male ② Female

5.Height: __cm Weight:______ kg

6.Ethnic groups: ① Han ② Ethnic minorities_________

7.Religion: ① None ② Yes_________

8.Frequency of participation in religious activities: ① once or twice a week ② three or four times a week

9.Marital status: ① unmarried ② married ③ widowed ④ divorced ⑤ other

10.Educational background: ① illiterate ② primary school ③ junior high school ④ senior high school and college education ⑤ bachelor's degree or above

11.Your past/ present occupation is: ① farmer ② teacher ③ worker ④ doctor ⑤ civil servant ⑥ freelancer ⑦ other

12. Your living style: ① live alone ② live with family/ friends ③ assisted institutions or nursing homes ④ others

13.Your residence: ① Town ② Countryside

14.Your monthly income is approximately: ①<1000 ②1000~ ③2000~ ④3000~ ⑤4000~ ⑥≥5000

15.Do you have financial pressure: ① No ② Yes

16.Your medical expenses: ① Cooperative medical care ② Self-paid ③ Public medical care ④ Commercial medical insurance ⑤ Others

17.Cancer types: ① lung cancer ② liver cancer ③ stomach cancer ④ colorectal cancer ⑤ breast cancer ⑥ others____

18.Have you had surgery: ① No ② Yes

19.Time from diagnosis to this survey:___________

20.Number of hospitalizations: ①1-3 ②4-6 ③7 or more

21.Have you had any adverse reactions during chemotherapy?

① No ② nausea/vomiting ③ anemia/bleeding ④ numbness of hands and feet ⑤ hair loss ⑥ others____

22.Have you participated in a clinical drug trial during your treatment: ① No ② Yes

23.Disease stage: ① I stage ② II stage ③ III stage ④ IV stage

24.Do you have any other diseases besides cancer: ① No ② Yes:__________

25. Knowledge of the condition: ① Do not know ② a little ② basic ② fully

26.Do you understand hospice care: ① Never understood ② only know a little ② understand and can explain

27. How you get information about hospice care: ① Magazines/books ② Mobile Internet ③ Other social media

Anxiety Care Perception and Need Scale

Note: Please draw on the number corresponding to the view you agree with most at the end of each entry✓ There is no right or wrong answer, and we are very interested in your current views.

When I hear about hospice care, how much do I agree with the following statement?							
	I couldn't agree more	disagree	Slightly disagree	I don't agree with it I don't object to that either	a little agree	agree	extraordinary agree
1. I was scared							
2. I feel hopeful							
3. I felt pressured							
4.I felt safe							
5. I'm frustrated							
6. I feel anxious							
7. I feel very comfortable							
If the doctor now suggests that I be referred to hospice care, I will...							
	I couldn't agree more	disagree	Slightly disagree	I don't agree with it I don't object to that either	a little agree	agree	extraordinary agree
8. I think the more support you get, the better							
9. I feel my illness is out of control							
10. I felt the doctor really cared about me							
11. I don't think there's anything else to try							
12. I felt I had reached the							

end of my life							
13. I felt like my doctor had given up on me							
14. I think my illness is terminal							
15. I feel like I'm going to lose touch with the doctors and nurses who are currently treating me							
16. I will look more positively at the future							
17. I feel like I'm in more control of my game							
18. I felt that there might be "someone from the unfamiliar hospice team" coming to my house							
19.I would worry they'd disrupt my daily life							
20.I would have feared they were going to talk to me about death							

21. Now let's learn about hospice care. Are you ready?										
0	1	2	3	4	5	6	7	8	9	10
Totally unprepared										Be fully prepared

The following needs fall within the scope of hospice care services and can help you, your family and friends. Please assess your current needs and status							
	I cou-ldn't agree more	disagree	Slightly disagree	I don't agree with it either I don't object to that either	Slightly agree	agree	extraordinary agree
22. My family or friends need to help me with phys-ical care							
23. I need help to control physical sym-ptoms like pain							
24. I need emotional support							
25. I need help with medicat-ion manag-ement							
26. I wanted to talk to people about death							
27. My family or friends need emoti-onal support							
28. I need spir-itual or emot-ional support							
29. I hope someone will talk to my family or fri-ends about my condition							
30. After gett-ing sick, I wa-nted to be							

helped to find meaning in my illness							
31. I want to be prepared for what might happen in the future							
32.I need someone to take care of my daily life, like taking a shower							
33.I wanted to talk to som-eone who co-uld underst-and what I was going through							
34. I hope my family and fr-iends will be prepared for what happens in the future							
How much would I agree with the following statement if I were to think about my current health?							
	I cou-ldn't agree more	disagree	Slightly disagree	I don't agree with it either I don't object to that either	a little agree	agree	extraordinary agree
35. I was wor-ried I'd be a burden to others							
36. I feel like I'm too depe-ndent on ot-hers to take care of me							
37.I worry that my family and friends don't have time for themselves because they're looking af-							

ter me							

Edmonton Symptom Evaluation System (ESAS)

Please circle the number that best describes your health status in the last 24 hours

Psychological Pain Thermometer (DT)

first ， Please rate the numbers on a scale that best matches your average level of distress over the past week✓ ”

No pain	0	1	2	3	4	5	6	7	8	9	10	It was excruciating
Never tired	0	1	2	3	4	5	6	7	8	9	10	Extremely tired
No nausea	0	1	2	3	4	5	6	7	8	9	10	It was extremely nauseating
No depression	0	1	2	3	4	5	6	7	8	9	10	Extremely depressed
Don't be anxious	0	1	2	3	4	5	6	7	8	9	10	HA
Don't be sleepy	0	1	2	3	4	5	6	7	8	9	10	Extremely sleepy
The appetite was excellent	0	1	2	3	4	5	6	7	8	9	10	Poor appetite
I feel the quality of life is excellent	0	1	2	3	4	5	6	7	8	9	10	I feel my quality of life is terrible
No itching	0	1	2	3	4	5	6	7	8	9	10	Extreme itching
No gas pressure	0	1	2	3	4	5	6	7	8	9	10	Extreme shortness of breath
Other issues	0	1	2	3	4	5	6	7	8	9	10	

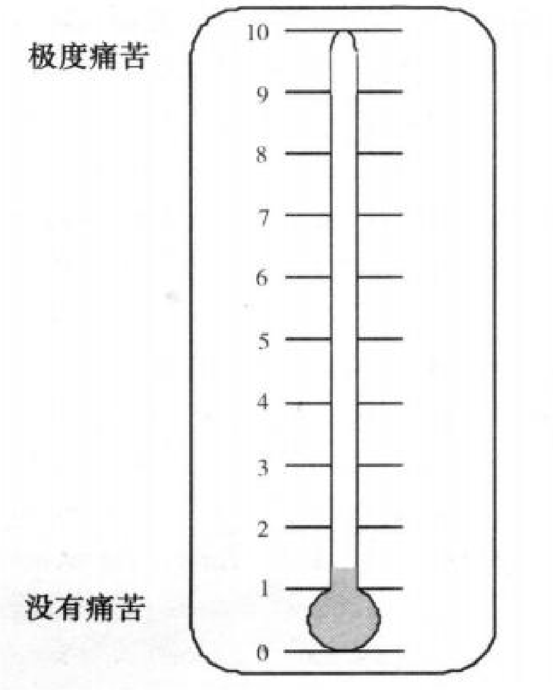

Next, indicate which of the following causes your distress? Check the item that applies✓ ”。

	physical problem
☐	No time and energy to take care of children/elderly
☐	No time or energy for housework
☐	economic issue
☐	transportation
☐	Work/study
☐	surrounding environment
	**Communication problems**
☐	Get along with children/old people
☐	Get along with your partner
☐	Get along with your friends
☐	Get along with the medical staff
	**Emotional problem**
☐	depressed
☐	fear
☐	lonely
☐	nervous
☐	sad
☐	worry
☐	Loss of interest in daily activities
☐	sleep
☐	Memory loss / difficulty concentrating

	physical problem
☐	Appearance/body
☐	Bath / Dressing
☐	breathe
☐	Changes in urination
☐	astriction
☐	diarrhoea
☐	take food
☐	weary
☐	hydroncus
☐	have a fever
☐	dizzy
☐	indigestion
☐	Oral pain
☐	feel like vomiting
☐	Nose dry / congested
☐	pain
☐	nature
☐	xerosis cutis
☐	Numbness in hands and feet
☐	Physical activity is limited
	**Faith/religion**
☐	Faith/religion
